# Contributions of DNA Damage to Alzheimer’s Disease

**DOI:** 10.3390/ijms21051666

**Published:** 2020-02-28

**Authors:** Xiaozeng Lin, Anil Kapoor, Yan Gu, Mathilda Jing Chow, Jingyi Peng, Kuncheng Zhao, Damu Tang

**Affiliations:** 1 Department of Medicine, McMaster University, Hamilton, ON L8S 4K1, Canada; linx36@mcmaster.ca (X.L.); yangu0220@gmail.com (Y.G.); mathildachow1994@gmail.com (M.J.C.); pengj31@mcmaster.ca (J.P.); kunchengzhao@icloud.com (K.Z.); 2 Urological Cancer Center for Research and Innovation (UCCRI), St Joseph’s Hospital, Hamilton, ON L8N 4A6, Canada; akapoor@mcmaster.ca; 3 The Research Institute of St Joe’s Hamilton, St Joseph’s Hospital, Hamilton, ON L8N 4A6, Canada; 4 Department of Surgery, McMaster University, Hamilton, ON L8S 4K1, Canada

**Keywords:** Alzheimer’s disease pathogenesis, diagnostic biomarkers for Alzheimer disease, DNA damage response, DNA damage repair

## Abstract

Alzheimer’s disease (AD) is the most common type of neurodegenerative disease. Its typical pathology consists of extracellular amyloid-β (Aβ) plaques and intracellular tau neurofibrillary tangles. Mutations in the *APP, PSEN1,* and *PSEN2* genes increase Aβ production and aggregation, and thus cause early onset or familial AD. Even with this strong genetic evidence, recent studies support AD to result from complex etiological alterations. Among them, aging is the strongest risk factor for the vast majority of AD cases: Sporadic late onset AD (LOAD). Accumulation of DNA damage is a well-established aging factor. In this regard, a large amount of evidence reveals DNA damage as a critical pathological cause of AD. Clinically, DNA damage is accumulated in brains of AD patients. Genetically, defects in DNA damage repair resulted from mutations in the *BRAC1* and other DNA damage repair genes occur in AD brain and facilitate the pathogenesis. Abnormalities in DNA damage repair can be used as diagnostic biomarkers for AD. In this review, we discuss the association, the causative potential, and the biomarker values of DNA damage in AD pathogenesis.

## 1. Introduction

Approximately 50 million people worldwide are affected by dementia (World Health Organization/WHO, 2019). With around 10 million new cases each year (WHO, https://www.who.int/news-room/fact-sheets/detail/dementia), dementia is estimated to affect 75.6 million and 135.5 million people worldwide by 2030 and 2050 (WHO) respectively. Among the neurodegenerative diseases that cause dementia, Alzheimer’s disease (AD) is the primary cause, accounting for 60%–70% of dementia cases (WHO). AD is a major health issue and the predominant burden on the healthcare system with respect to dementia.

AD was first described by Alois Alzheimer more than one century ago on a patient named Auguste D [1]; the typical pathological features include extensive cerebral amyloid plaques and neurofibrillary tangles [2,3]. The latter is caused by intraneuronal aggregation of hyperphosphorylated tau; abnormal tau phosphorylation is attributable to several kinases including CDK5 [4,5,6,7]. Additionally, CDK5 phosphorylates the amyloid precursor protein (APP) at threonine 668 (T668), which stimulates β-amyloid (Aβ) peptide accumulation [8]. Aggregation of Aβ peptides, particularly Aβ_1-42_, causes amyloid (senile) plaques in AD brain [9,10,11]. Furthermore, CDK5 activities affect DNA damage response [12], supporting a linkage of DNA damage with AD [13,14].

Aβ peptides are directly produced by sequential cleavages of APP by β- and γ-secretase; the proteolytic c-terminal fragment of APP (CTFβ) of β-secretase is cleaved within the cell membrane by γ-secretase to generate neurotoxic Aβ peptides with length ranging from 38-43 residues [15,16,17,18,19]. The 40 residue Aβ peptide (Aβ_1-40_) is the major product, accounting for more than 50% of Aβ peptides [9,17]. Although the Aβ_1-42_ peptide consists of less than 10% of Aβ peptides [16,17], it is more neurotoxic and associates with a higher risk of AD because of its propensity of aggregation [9]. The importance of Aβ in AD pathogenesis is illustrated by mutations of *APP, PSEN1,* and *PREN2* genes in familial AD with the latter two encoding the presenilin 1 and presenilin 2 subunit of γ-secretase. Individuals with these mutations develop early onset dementia in an autosomal-dominant manner [20]; the typical onset starts between 30 and 50 years of age in *PSEN1* mutant carriers with some being affected at in their 20s [20,21]. γ-secretase with mutant presenilin 1 or presenilin 2 subunit favors Aβ_1-42_ production [16,17]. These genetic observations led to formation of the amyloid cascade hypothesis, in which abnormal Aβ drives AD pathogenesis via regulating other pathological events including tau pathology [22,23,24,25,26,27]. This hypothesis is supported by the similar pathological features between familial AD and sporadic late onset AD (LOAD) [20]. Although familial AD constitutes less than 1% of AD cases [28,29], the hypothesis has been widely accepted to guide research in advancing the understanding of both familial AD and LOAD in the past two decades [30,31].

**Figure 1:**
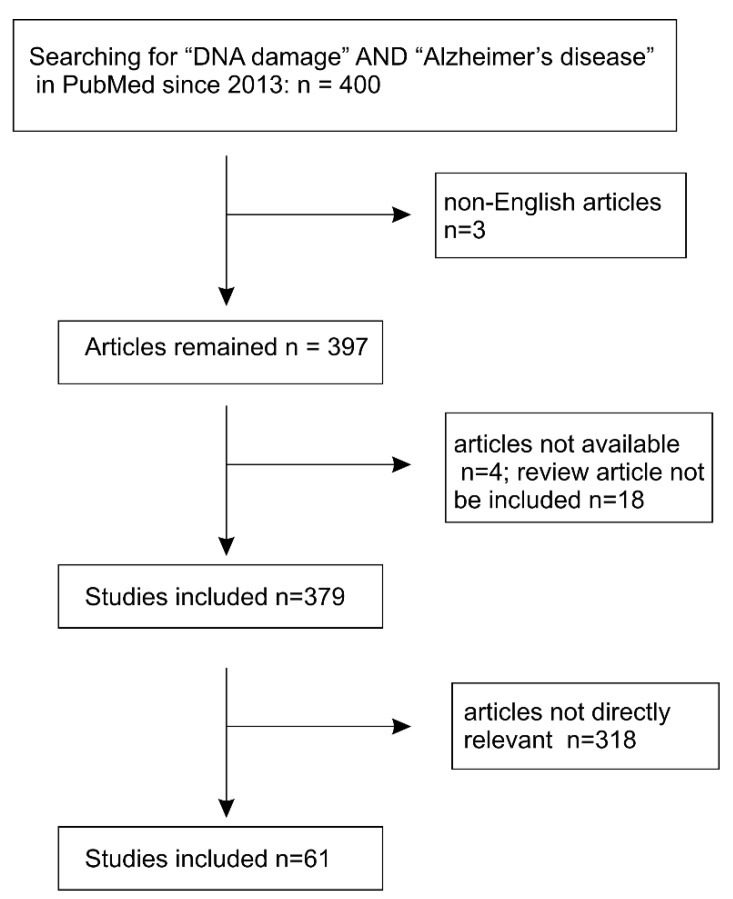
Systemic literature search and selection of articles for review.

Nonetheless, it is becoming increasingly clear that complex pathological etiology instead of the amyloid-cascade hypothesis is involved in LOAD, which accounts for more than 90% of AD cases in patients over 65 year old [32,33]. This concept is supported by the lack of success in all phase III clinical trials on drugs developed to remove cerebral Aβ in attempt to slow down cognitive decline in patients with either mild cognitive impairment (MCI) or AD dementia [31,34]. In sporadic LOAD cases, aging is the strongest risk factor [30,35]. Aging is clearly a complex process; nonetheless, accumulative evidence reveals a central role of DNA damage in aging [36]. Of note, DNA damage is clearly linked to AD and other neurodegenerative diseases. The topic of “DNA and neurodegenerative diseases” has been previously reviewed [14,30,37]. We will update the recent developments from 2014 onward; key publications prior to 2014 will be covered. Data used in this review were extracted from PubMed and selected according to the PRISMA Guidelines [38,39] (Figure 1).

## 2. Association of DNA Damage with Alzheimer’s Disease

The association of DNA damage with aging is well studied and established with 7086 articles under “DNA damage” AND “aging” listed in PubMed as of January 17, 2020. Genome rearrangement and double strand breaks (DSBs) increase in aging mice and senescing human cells [40,41]. DNA lesions are accumulated in AD brains. Elevations in γH2AX, a well-established marker of DSB [42], were detected in 11 of 13 AD brains in astrocytes of hippocampus and cerebral cortex [43]. Using two independent cohorts (*n* = 13 and *n* = 23), significant increases in γH2AX were recently demonstrated in astrocytes and neurons in the hippocampus and frontal cortex of AD brains [44]; the increase occurred in brains with mild cognitive impairment (MCI) [44], a preclinical stage of AD [45,46], suggesting an important role of DSB in AD pathogenesis. Furthermore, DSBs and single strand breaks (SSBs) were demonstrated at the DNA level in hippocampi of AD brains [47].

The detection of both DSBs and SSBs in AD brains supports the knowledge that endogenous reactive oxygen species (ROS) is the major source of DNA damage in AD brains [48], considering the brain being protected from external or environmental genotoxins through the blood–brain barrier. Post-mitotic neurons of the central nervous system have a high rate of metabolism [49]; the human brain accounts for 2% of body mass and consumes 20% of oxygen [50]. In this regard, accumulation of DNA oxidization was observed in AD brains [51,52]; the lesion was also detected in brains with MCI [53,54,55] and preclinical AD (PCAD) [55,56]. PCAD subjects are clinically normal, i.e., without overt manifestation of AD, but with pathological features of AD [56,57]. The proceeding of oxidative DNA lesions in MCI and PCAD conditions supports a causative contribution of DNA damage induced by ROS in AD. The predominant base adduct of DNA lesion caused by ROS is 8-hydroxyguanine (8-OHG); 8-hydroxy-2’-deoxyguanosine (8-OHdG) is a widely used marker of DNA oxidation. Increase of 8-OHdG in ventricular cerebrospinal fluid (CSF) was observed in AD brains, suggesting a biomarker value of 8-OHdG in AD diagnosis [58].

Collectively, evidence suggests a role of DNA damage with respect to DNA oxidation and the resultant DSBs and SSBs in AD pathogenesis. The accumulation of these DNA lesions is in part attributable to reductions in the repair of these DNA damages. By using autoradiographic methods to examine DNA damage (SSB) and repair capacity in neurons, an inverse association of accumulation of nuclear DNA (nDNA) breaks and nDNA repair was documented in aging mouse brain [59,60]; accumulation of nDNA SSB was detected in mouse cerebellar granule cells as well as hippocampal pyramidal and granule cells [61], which underlines a causative role of DNA damage in AD pathogenesis.

## 3. Link of DSB with AD

Brain is particularly vulnerable to impairment in DNA repair [62], which is likely attributable to the high levels of metabolism and transcription activities in neurons of the central nervous system (CNS) [48,49,63]. In addition to ROS as a source of DSBs in neurons of CSN, recent development reveals an essential association of transcription with DSB [64]. Stimulation or performance of variety of physiological tasks generate DSBs in neurons [63,65,66]. DSBs occur in the promoters of several early response genes and contribute to their transcription [65]; early response genes function in learning and memory [67,68] suggesting an association of DSB accumulation with cognitive decline, a major defect in patients with AD. Consistent with these observations, the number of DSB was increased in transgenic mice of hAPP (human APP mutant) [63]. While the underlying mechanisms responsible for DSB generation in neurons performing physiological activities remain to be determined, it is likely that topoisomerase IIβ (TOP2β) produces these DSBs [65,69,70,71,72]. It was first reported by Ju et al. in 2006 that DSB generation by the TOP2β-PARP1 complex in the pS2 promoter is required for estrogen-induced transcription of the target gene in MCF7 cells [69]. Subsequently, glucocorticoid receptor was also reported to transactivate genes via TOP2β-produced DSBs [73]. Additionally, TOP2β is expressed in differentiation cells and neurons [74,75]; the expression of long-transcripts linked to autism is facilitated by TOP2β [76]. While it is indeed intriguing for an essential role of DSBs at least in a subset of gene transcription, it is clear that DSBs need to be effectively repaired regardless of their sources of generation owning to their toxic impact on genome stability [77]. Reduction in DSB repair will lead to DSB accumulation, neuron loss, cognitive decline, and thus AD [44,78,79].

**Figure 2:**
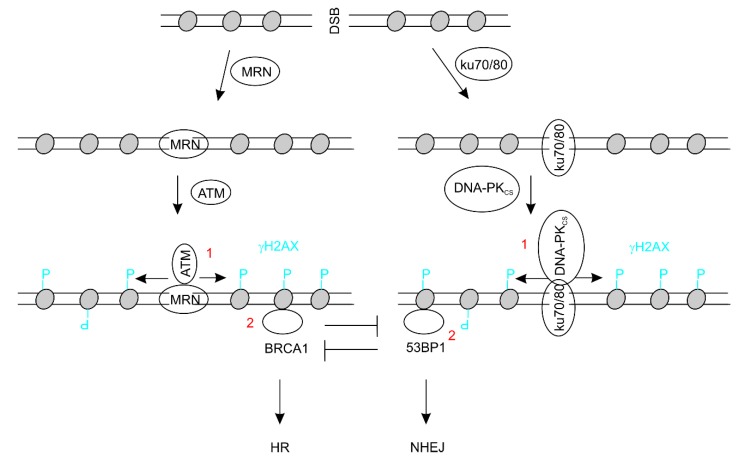
A model showing double strand break (DSB) repair pathways. For the homologous recombination (HR) and non-homologous end joining (NHEJ) pathways, DSBs are first recognized by either the MRN or Ku70/80 complex, followed by the recruitment of ATM or DNA-PK_CS_ (the catalytic subunit of DNA-PK) as indicated. ATM and DNA-PK then phosphorylate H2AX at serine 139 to generate γH2AX (event 1), which initiates event 2: recruiting either BRCA1 or 53BP1; recruitment of either inhibits the recruitment of another. 53BP1: p53-binding protein 1; ATM: ataxia-telangiectasia mutated; BRCA1: breast cancer type 1 susceptibility protein; DSB: double strand DNA break; DNA-PK: DNA-dependent protein kinase; HR: homologous recombination; MRN: the complex of MRE11-NBS1-RAD50; NHEJ: non-homologous end joining.

DSBs in mammalian cells are repaired by either homologous recombination (HR) or non-homologous end joining (NHEJ) [80]. DSBs result in activation of ATM (ataxia-telangiectasia mutated) and DNA-PK (DNA-dependent protein kinase) kinases which play essential roles in DSB repair (Figure 2) [81,82,83]. Recruiting ATM to DSB is mediated by the MRN complex; ATM action leads to BRCA1 recruitment, a commitment step for utilization of HR to repair DSBs (Figure 2) [80,84,85]. On the other hand, DSBs also recruit DNA-PK though the Ku70/Ku80 complex, followed by 53BP1 recruitment, which commits to NHEJ-mediated DSBs repair (Figure 2) [86]. The recruitment of BRCA1 and 53BP1 are mutually exclusive (Figure 2).

### 3.1. Association of HR in AD

Evidence suggests a contribution of decreases in HR to AD. ATM is the apical kinase in mediating DSB repair by HR (Figure 2). Ataxia telangiectasia (A−T) is caused by mutations in the *ATM* gene; the disease manifests defects in multiple system including its typical clinical presentation of ataxia due to progressive neurodegeneration that occurs in early childhood [87]. In comparison to age-matched control subjects, reductions in both the ATM protein and its mRNA were detected in the front cortex of AD brains (*n* = 9, 5 males and 4 females) [88]. Downregulation of ATM was also suggested in 3 mouse models for AD, transgenic mice expressing mutants of *APP*, *PSEN1/APP*, or *PSEN1/APP/MAPT* (encoding tau) [88].

BRCA1 plays an essential role in DSB repair using the HR process and recruitment of BRCA1 to DSBs commits cells to HR (Figure 2) [80,86]. Reductions in BRCA1’s ability in managing DSB repair are suggested in AD brains. Decreases in BRCA1 expression were detected in the hippocampal neurons of not only AD but also MCI brains [89], indicating a causative role of BRCA1 in AD. In this respect, transgenic expression of hAPP in mice leads to decrease in BRCA1 expression; knockdown of BRCA1 in these mice increases neuronal DSBs and apoptosis with concurrent impairment of learning and memory [89]. On the other hand, exploratory activities induced by change in environment upregulate BRCA1 in the dentate gyrus of mouse hippocampus [89]. These results are well in accordance with the concept that physiological neuronal activities increase transcription of early response genes through processes promoted by DSBs [65]. It is thus intriguing to envisage a role of BRCA1 in the repair of those transcription-utilized DSBs. This possibility is further supported by induction of the HR protein RAD52 by nascent mRNAs in terminally differentiated neurons and Aβ inhibits RAD52 expression [90]. In the *APP/PSEN1* mouse model for AD, a decrease in the HR protein RAD51 co-exists with DSB accumulation [91]. Additionally, the *BRCA1* promoter is hypomethylated and the BRCA1 protein is increased in AD brains; however, the protein was found to be mis-located into the cytoplasm as a highly non-soluble protein through association with tau; BRCA1 in AD brains is thus dysfunctional with respect to DSB repair [92]. This BRCA1 dysfunctionality can be directly induced by Aβ peptides [92]. The association of BRCA1 with phosphorylated tau in AD brain was also observed [93], consistent with tau hyperphosphorylation causing tau aggregation. In neurons differentiated from induced pluripotent stem cells (iPSC) that were reprogrammed from fibroblasts of familial AD patients, location of BRCA1 to cytosol is also demonstrated [89]. Collectively, evidence favors a critical role of BRCA1 in DSB repair in CNS neurons and its dysfunction contributes to DSB accumulation and AD pathogenesis.

The ATM-BRCA1 pathway of HR not only plays a role in AD through the management of DSB repair but also may affect AD indirectly. FE65 is an adaptor protein with its expression enriched in the brain [94,95]. It binds the APP intracellular domain (AICD), the c-terminal fragment of Aβ, contributes to ACID-derived transcription activities in mice, and likely plays a role in AD [94,96]. FE65 interacts with Tip60 and has an important role in DNA damage response (DDR) in SK-N-SH neuroblastoma cells [97]. Tip60 has a key function in DSB-induced ATM activation through acetylation of ATM at lysine 3016 [98,99]; mice deficient in FE65 are more sensitive to DNA damage [96]. Evidence thus supports a connection between FE65 and ATM in DSB repair in neurons. This concept is supported by recent discoveries of FE65 being an ATM target during DDR [100,101].

While evidence supports a role of HR in the repair of DSBs in post-mitotic neurons, many issues remain unclear. HR requires DNA replication and thus takes place in late S and G2 phase [80,86]; the mechanisms underlying HR in DSB repair of terminally differentiated neurons are still unclear. In studies using neurons differentiated from iPSCs reprogrammed from patients with familial AD, ATM and BRCA1 signaling were found to associate with the expression of cell cycle elements [88,89]; this suggests cell cycle re-entry as a potential mechanism for utilization of HR in post-mitotic neurons. Nonetheless, more evidence is needed to uncover the full mechanism at play.

### 3.2. Link of NHEJ in AD

NHEJ makes a major contribution to DSB repair because it does not rely on cell division and is the primary choice for differentiated cells to repair DSBs [80,86]. In this regard, downregulation of NHEJ is likely a contributor to DSB accumulation in the AD brain. Herpes simplex virus-type 1 (HSV-1) infection is a well-recognized risk factor of AD [102,103]. HSV-1 induces AD via affecting multiple AD events, such as through enhancing Aβ_1−42_ production [104]. The virus causes DSB accumulation in rat cortical neuron through downregulation of Ku80, an essential component of NHEJ (Figure 2), and thus impairs NHEJ in cortical neurons [105].

DNA-PK consists of the Ku70/Ku80 subunits and the catalytic subunit DNA-PKcs [106,107]. In a retrospective study using post-mortem AD brains, reductions in both Ku and DNA-PKcs in cortex were suggested [108]. However, reductions in DNA-PK expression did not reach a level of statistical significance [109]. In immunodeficient mice lacking DNA-PK, hippocampal CA1 and CA3 neurons are vulnerable to DNA damage [110]. In vitro, Aβ peptides including Aβ_1−42_ reduce DNA-PKcs expression and compromises DSB repair in PC12 cells [111].

In consideration of the unique characteristics of NHEJ [80,86], reductions in the DNA-PK-dependent classical NHEJ (c-NHEJ) are conceptually the primary cause of DSB accumulation in AD brains in comparison to HR. Additionally, recent evidence shows DSB generation to be an essential process in transcription initiation [69] and elongation [112] in which DSBs are managed by TOP2β complexed with DNA-PK [69]; the involvement of DNA-PK in the process is likely for dynamic repair of DSBs. As high transcription activity in post-mitotic neurons remains a major source of DSBs associated with neuron activities and transcription [63,65,66], it seems logical for defects in NHEJ being favored over HR leading to DSB accumulation in AD. Nonetheless, the reverse seems evident based on the available studies (see Section 3.1). Furthermore, in a direct comparison study using *APP/PSEN1* transgenic mice, reductions in RAD51 but not 53 BP1 expression are evident in the hippocampus of both young and aging mice [91]. RAD51 and 53BP1 are essential in HR and NHEJ respectively (Figure 2). Evidence thus supports a major contribution of HR defects in DSB accumulation in AD.

## 4. Defects in Base Excision Repair (BER) in AD

**Figure 3:**
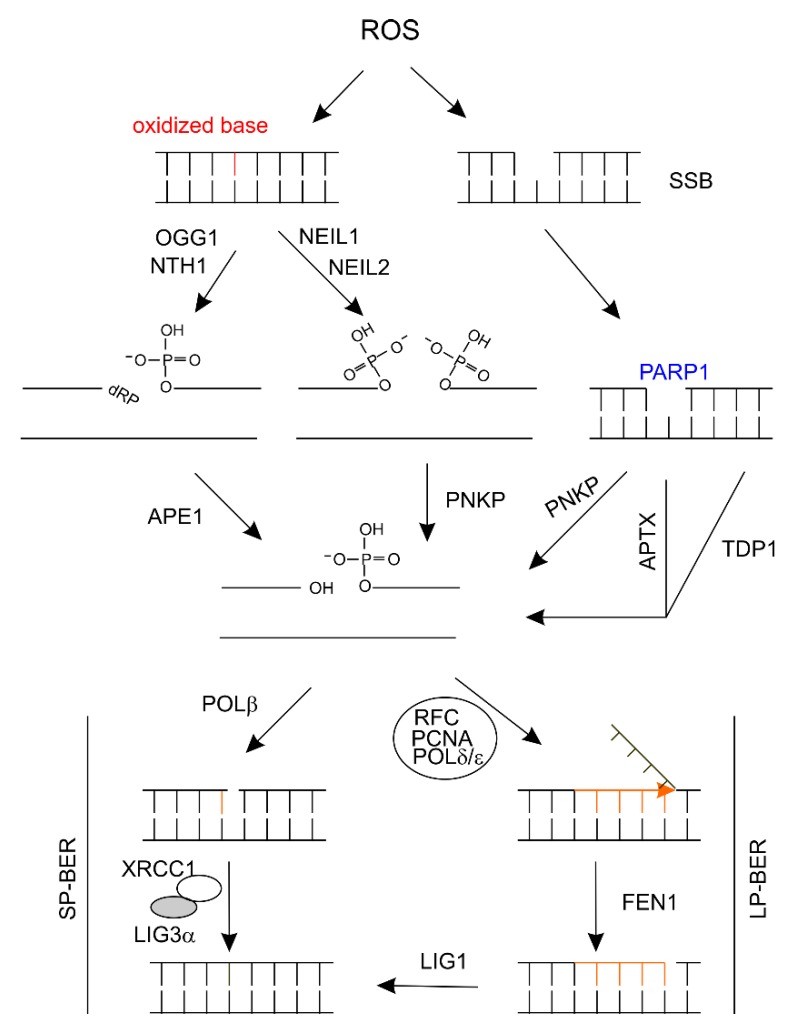
An illustration demonstrating BER. ROS induces oxidized base lesion or SSB. The oxidized bases are removed by DNA glycosylase OGG1 and NHT1 or NEIL1 and NEIL2; the ends are then processed, followed by filling the gap with synthesis of a single nucleotide or 2-8 nucleotides; ligation via a ligase will then complete the repair. SSB was first recognized by PARP1; different ends produced by end processing are accordingly modified by the indicated proteins, followed by gap filling using either the SP-BER or the LP-BER pathway. APE-1: AP (apurine/apirimidine) endonuclease 1; APTX: ataxia with oculomotor apraxia; BER: base excision repair; dRP: 3’ phosphor-α,β-unsaturated aldehyde; FEN1: Figure 1. LIG: DNA ligase; NEIL1: Nei like DNA glycosylase 1; NEIL2: Nei like DNA glycosylase 2; NTH1: Nth like DNA glycosylase; OGG1: DNA glycosylase; PARP1: poly(ADP) ribose polymerase 1; PNKP: polynucleotide kinase phosphatase; POLβ: DNA polymerase β; ROS: reactive oxygen species; SSB: single strand DNA break; TDP1: tyrosyl-DNA phosphodiesterase 1; XRCC1: X-ray repair cross-complementing protein 1.

Cells are more prone to SSB compared to DSB; mammalian cells are estimated to have approximately 10^4^ oxidized base and SSB [113] in comparison to approximately 50 DSBs daily [114]. This concept is supported by a large amount of evidence revealing ROS as a major source of DNA damage in terminally differentiated neurons of CNS [48]. Oxidized bases are repaired via BER [49]. Repair starts with the recognition and removal of an oxidized base by a DNA glycosylase, including 8-oxoguanine (8-oxoG) DNA glycosylase/OGG1, NHT1, NEIL1, or NEIL2 (Figure 3). The end products are modified by AP (apurine/apirimidine) endonuclease 1 (APE-1) or polynucleotide kinase phosphatase (PNKP) to make the ends competent for DNA synthesis (Figure 3). The gap is filled by DNA polymerase β (POLβ), followed by ligation with DNA ligase IIIα (LIG3α) with assistance from the X-ray repair cross-complementing protein 1 (XRCC1) (Figure 3); this process of gap filling by incorporation of single nucleotide is known as short-patch BER (SP-BER). Alternatively, the gap is filled by long-patch BER (LP-BER) with the incorporation of 2-8 nucleotides (Figure 3). ROS can produce SSB, which is recognized by poly(ADP) ribose polymerase 1 (PARP1); the ends are then processed by PNKP, ataxia with oculomotor apraxia (APTX), or tyrosyl-DNA phosphodiesterase 1 (TDP1), followed by gap filling with either SP- or LP-BER (Figure 3) [49,115,116,117].

### 4.1. Reductions of DNA Glycosylase Activity in AD

Decreases in 8-oxoguanine (8-oxoG) DNA glycosylase activities occur in the neurons of hippocampus and other regions of AD brains (*n* = 10) compared to age-matched brains (*n* = 8) [118]. Reductions in DNA glycosylase activities and POLβ expression were also observed in AD brains (*n* = 10) in comparison to age-matched control brains (*n* = 10) [119]. Downregulation of mitochondrial BER activity in AD brain was also reported [120]. Furthermore, impairment of BER also occurs in MCI brains (*n* = 9) [119], indicating an important role of BER capacity decreases in AD pathogenesis.

Deletion of codon C796 of OGG1 was detected in 2 AD brains out of 14 samples; protein encoded by the deletion leads to complete loss of 8-oxoG DNA glycosylase activity [121]. Mutations leading to change of alanine 53 to threonine (A53T) and A288V (valine) were detected in AD brains [121]; the substitutions result in significant decreases in 8-oxoG DNA glycosylase activity (Table 1) [121]. Both A53T and A288V variants decrease interaction with PARP1 and XRCC1; while A53T reduces binding to substrates, A288V compromises AP lyase activity [122]. Significant decreases of the NEIL1 DNA glycosylase protein expression were detected in AD brains (n = 6) compared to control brains (*n* = 6) [120]. NEIL1 null mice are defective in memory retention in a water maze test [123]. NEIL1 and NEIL2 are members of the Nei family of DNA glycosylase [124,125,126,127]; their expressions are increased during rat brain development [128], suggesting the importance of NEIL1 and NEIL2 in maintaining genome integrity of CNS. Taken together, evidence supports that declines in NEIL1 expression in brain facilitate AD.

### 4.2. Reductions of PARP1 Activity in AD

The impact of PARP1 on AD is complex. By ADP-ribosylation of target proteins though utilization of nicotinamide adenine dinucleotide+ (NDP+), PARP1 regulates metabolism; persistent PARP1 activation results in depletion of cellular NDP+, which leads to decreases in ATP production and alterations in other cellular events including induction of apoptosis [130,131]. Aβ peptides activate PARP1 via induction of oxidative stress in the hippocampus of adult rats [132], which likely contributes to AD via affecting brain metabolism [133]. Elevations in PARP1 activity and increases of PAR level in neurons were observed in AD brains compared to controls (Table 1) [129,133]. Evidence suggests a toxic impact of high levels of PARP1 activity in part through NAD+ depletion, as administration of exogenous NAD+ reduces Aβ-caused toxic effects in primary rat cortical neurons [134]. Concurrently, addition of NAD+ also protects neurons from Aβ-induced DNA damage [134], consistent with the concept that defects in PARP1-derived DNA repair contribute to AD. Of note, decreases in nucleolar PARP1 in hippocampal neurons of AD brains (*n* = 8) were recently reported [135]. Evidence thus indicates an insufficient level of NAD+ in AD brains as a cause of PARP1 dysfunction. In the 3xTgAD (transgenic co-expression of the Swedish APP mutations KM670/671NL, PSEN1 mutation M146V, and Tau mutation P301L) mice, reductions of NAD+ in cerebrum occur. In these AD mice, normalization of NAD+ by nicotinamide riboside (NR) treatment attenuate the Tau pathology but not Aβ accumulation with concurrent reductions in DNA damage and improvement of cognitive function [136]. These benefits are greater in 3xTgAD/POLβ+/− mice than in 3xTgAD mice [136], supporting that the NR treatment improves BER. Additionally, Aβ_23−35_ and Aβ_1−42_ increase oxidative DNA damage, SSB, and DSB in rat cortical neurons in vitro and addition of nicotinamide adenine dinucleotide (NAD) reduces all these toxic events [134]. Collectively, these studies highlight a possible clinical management of AD patients using NAD-related approaches. Polymorphisms of PARP1 are associated with AD risk [137,138]; nonetheless, the impact of these polymorphisms on PARP1 activity remains unclear.

### 4.3. A Major Contribution of Decreases in POLβ to AD

POLβ plays a role in AD [119]. The protein is expressed at reduced levels during aging in multiple tissues, including brain, kidney, liver, spleen, and testes in mice [139] and in the aging neurons of rat [140]. Loss of POLβ leads to senescence, indicating a major role of POLβ in anti-aging [141]. This concept is supported by a major contribution of defects in BER caused by POLβ reduction to Down syndrome (DS) [142,143], a typical process of precocious aging [144]. These observations comprehensively support a critical role of POLβ in regulating aging progression. As aging is the strongest risk factor of AD, it is conceivable that POLβ may play an important role in AD. This hypothesis is in accordance with the observation that almost all patients with DS developing AD pathology by their 40s [145,146,147,148].

Decreases in DNA synthesis-dependent gap filling activities occur in MCI and AD brains, which is in part attributable to a significant reduction of POLβ expression (Table 1) [119]. Mice with genetic downregulation of POLβ (POLβ+/−) in the 3xTgAD background display neuron death and worsen memory and synaptic plasticity with concurrent increases in DNA damage in comparison to age-matched 3xTgAD mice [149]. In comparison to 3xTgAD mice, 3xTgAD/ POLβ+/−mice exacerbate olfactory deficit in part via attenuation of neuron generation by neural progenitor cells [150].

While these data clearly reveal a critical role of POLβ in AD pathogenesis, reduction of POLβ alone is not sufficient to initiate AD; POLβ+/− mice express POLβ at a comparable level at 6 month old and a reduced level at 14 month old compared to 3xTgAD mice, the latter genotype mice develop AD pathology [149].

### 4.4. Coordination of BER Defects in AD

**Figure 4:**
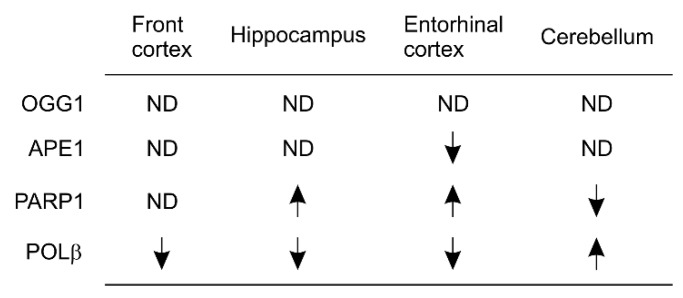
Summary of expression of the indicated BER genes in AD brains compared to age-matched controls. Arrows indicate upregulation and downregulation respectively. APE-1: AP (apurine/apirimidine) endonuclease 1; ND: no differences; OGG1: DNA glycosylase; PARP1: poly(ADP) ribose polymerase 1; and POLβ: DNA polymerase β.

The high rate of metabolism and oxygen utilization as well as a low ratio of antioxidant to pro-oxidant enzymes in post-mitotic neurons of CNS [48] make these cells rely on BER to manage oxidative DNA damage. This knowledge agrees with an important contribution of BER capacity reductions to AD pathogenesis. However, it seems not all major components of BER as illustrated in Figure 3 are clearly affected in AD. For instance, the polymorphism of R194W (arginine 194 to tryptophan 194) of XRCC1 does not appear to be a significant risk factor of AD [151,152]. While reagent limitations restrained the detection of APE1 in post-mortem brain by immunohistochemistry [153], the APE1 mRNA level is decreased in entorhinal cortex in AD brains compared to controls (Figure 4) [154]. It thus remains to be determined whether BER dysfunction in AD is resulted through a coordinated manner. A recent report has systemically determined the expression of OGG1, APE1, PARP1, and POLβ in a cohort of 42 ADs and 9 controls using quantitative real-time PCR [154]. Consistent with the discussions in Section 4.2, PARP1 mRNA expression is increased in hippocampus and entorhinal cortex (Figure 4) [154]; while APE1 mRNA is only reduced in entorhinal cortex, POLβ is significantly reduced in frontal cortex, hippocampus, and entorhinal cortex (Figure 4) [154], suggesting a possibility for alterations in more than one factor to impair BER during AD pathogenesis.

## 5. Other AD Risk Factors Affecting DNA Repair

### 5.1. Role of CDK5 Abnormalities in AD Via Affecting DNA Damage

Unlike of majority of the cyclin-dependent kinase (CDK) family, CDK5 is most well-studied for its neuron-specific functions owing to the identification of its neuron-specific activators p35 and p39 in 1994 and 1995 [155,156,157]. Accumulative studies in the past 2 decades established essential roles of CDK5 in CNS development in mice, including synaptic plasticity and memory [158], the major neuronal functions that are compromised in AD. In this regard, abnormalities in CDK5 activation play major roles in AD pathogenesis [159]. In response to increases in calcium concentration, p35 is cleaved to p25 which is more stable and causes CDK5 hyper-activation; additionally, CDK5/p25 complex has changes in substrate specificity and cellular localization [160]. Abnormal CDK5 activity promotes the major AD pathology: Extracellular senile plaque and intracellular neurofibrillary tangles (NFTs) through facilitating Aβ production and tau phosphorylation [7,159,161,162,163], and thus impairs synaptic plasticity and induces neuronal cell death [159]. Abnormal CDK5 activation leads to ROS accumulation in neuronal cells including Neuro-2a cells [164,165], suggesting a role of CDK5 dysfunction in DNA damage in neurons. Of note, induction of transgenic expression of p25 in postnatal mouse forebrain results in AD progression, Aβ accumulation, tau neurofibrillary tangles, synaptic density reduction, neuron loss, and accumulation of DSBs [8,166].

**Figure 5:**
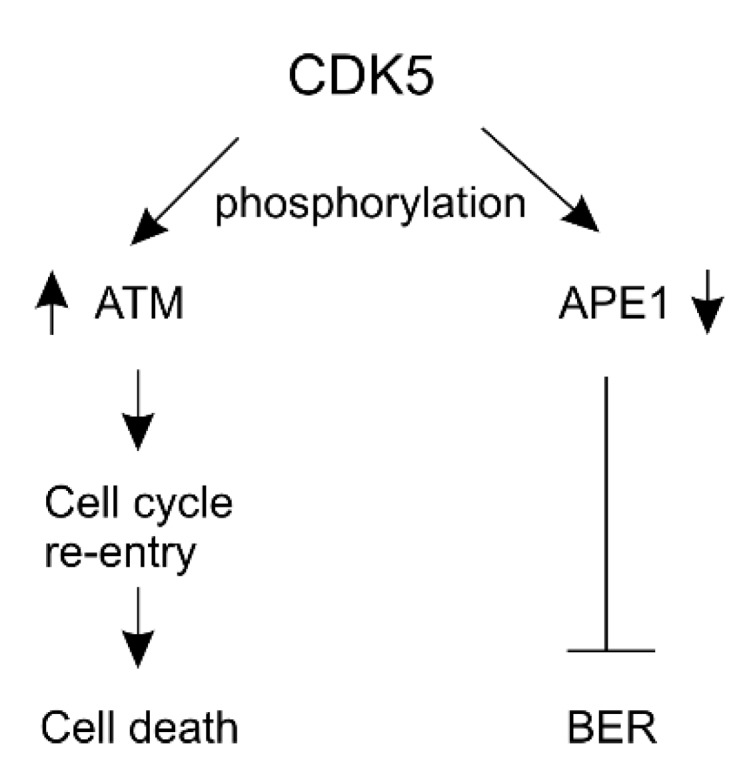
An illustration shows the effects of CDK5 in regulating DNA repair in neurons. Arrows indicate enhancing (upward direction) and reducing (downward direction) the protein’s functions respectively. CDK5 activity towards ATM in neurons is likely via p25. APE-1: AP (apurine/apirimidine) endonuclease 1; ATM: ataxia-telangiectasia mutated; BER: base excision repair; CDK5: cyclin-dependent kinase 5.

Mechanistically, CDK5 contributes to DNA damage in neurons likely via multiple pathways. In line with HSV-1 infection as a well-recognized risk factor of AD [102,103] in part via inducing DSB accumulation in neurons [105], the virus stimulates CDK5 activation, changes its subcellular location, and induces γH2AX nuclear foci (DSB marker) in infected mouse neurons [167]. CDK5 facilitates DSB-induced DDR via enhancing ATM activation in primary cerebellar granule neurons isolated from rats (Figure 5) [168]. How this action contributes to DSB accumulation remains unclear, as ATM activation is required for HR-mediated repair of DSBs. Nonetheless, CDK5 induces neuronal cell death partly via ATM activation [168] (Figure 5) and the connection of CDK5/p25-ATM is a cause of DDR-induced neurodegeneration in a mouse model for fragile-X-associated tremor/ataxia syndrome (FXTAS) [169]. Protein phosphatase 4 (PP4) dephosphorylates 53BP1 in late mitosis, an event required for 53BP1 recruitment to DSB in G1 phase for NHEJ-mediated repair of DSBs. CDK5 phosphorylates PP4R3β, the PP4 regulatory subunit, which facilitates PP4 action and contributes to cell proliferation-associated NHEJ [170]. However whether this function is active in post-mitotic neurons remains to be determined.

CDK5 reduces BER in neurons. CDK5 interacts with APE1 and phosphorylates it at threonine 232 (T232), which reduces APE1’s activity in cleavage of abasic (AP) sites and thus inhibits BER in mouse cortical neurons (Figure 5) [171].

### 5.2. Downregulation of Sirtuine 6 (SIRT6) Facilitating AD in Part Via Decreases in DNA Repair

SIRT6 is an emerging risk factor of AD [172] with 12 articles listed in PubMed under “SIRT6” AND “Alzheimer’s disease” (January 22, 2020). SIRT6 is a longevity gene [172,173]. The anti-aging activities of SIRT6 can be attributable to its NAD+ dependent histone deacetylase activity that functions in energy metabolism, inflammation, telomere maintenance, genome integrity and DNA repair [172,174,175,176,177,178]. SIRT6 null mice display genome instability and premature aging [176]. Mice with brain-specific SIRT6 deficiency showed impaired learning by the age of 4 months, elevated DNA damage, and development of tau phosphorylation-dependent toxicity [179]. Importantly, SIRT6 expression is significantly reduced at the protein level in AD brains (*n* = 7) compared to controls (*n* = 7) [179,180]; at the mRNA level, decreases in SIRT6 expression are associated with AD progression from Braak stage iii (*n* = 11) to v (*n* = 11) or vi (*n* = 23) [179]. In a cohort consisting of postmortem AD brains (*n* = 32) and control subjects (*n* = 47), reductions of SIRT6 occur in temporal cortex and hippocampus [179]. In the 5XFAD mouse model for AD [expressing three APP mutants (Swedish K670N/M671L, Florida I716V, and London V717I) and 2 PSEN1 (M146L and L286V) mutants], SIRT6 expression is reduced in hippocampus and frontal cortex [180]. Aβ42 reduces SIRT6 expression and induces DNA damage in mouse hippocampal neurons; overexpression of SIRT6 prevents the DNA damage [180]. SIRT6 contributes to BER and DSB repair [172]. SIRT6-mediated protection of neuron injury is inhibited by miR-34a-derived SIRT6 downregulation [181]. Collectively, evidence reveals SIRT6 to show protective effect for AD in part via enhancing DNA repair. Intriguingly, SIRT6 was reported to promote hippocampal neurogenesis in SIRT6 overexpressing adult mice [182], implying a role of SIRT6 in facilitating progenitor-derived neurogenesis in part via repairing oxidative stress-induced DNA damage.

### 5.3. A role of DNA Damage in AD Via Affecting Neurogenesis

Neurons of CNS are regenerated in the adult brain via a process that is mediated by neural stem cells (NSCs); compromising neurogenesis is a cause of neurodegeneration diseases, including AD [183,184]. Oxidative DNA damage and its repairing process affect NSC proliferation and differentiation [184]. For instance, SIRT6 exerts anti-AD function in part via facilitating neurogenesis of hippocampal neurons via enhancing BER and DSB repair [182]. Maintenance of the stemness of NSC requires BMI1 (B lymphoma Mo-MLV insertion region 1) [185,186,187,188,189,190]; the process is partly mediated by suppression of the INK4A/ARF locus [191], which encodes two important tumor suppressors p16INK4A and p14ARF (or p19ARF in mice) [192,193]. BMI1 is emerging to facilitate DSB repair through both the HR and NHEJ pathways [194,195,196,197]. BMI1 protein expression is reduced in post-mortem AD brains (*n* = 2) in comparison to age-matched controls (*n* = 2) [198]; the downregulation is detected in LOAD brain but not in early-onset familial AD (FAD) [199]. BMI1 heterozygous mice develop cognitive deficiency with accumulation of tau phosphorylation, Aβ plaques, and neuron loss [198]; the animals also display reductions in DDR [198]. Additionally, knockout of BMI1 in post-mitotic neurons induces Aβ deposition and tau hyperphosphorylation [199]. Collectively, evidence suggests a role of BMI1 in reducing AD via facilitating neurogenesis partially through DNA repair.

BRCA1 plays an essential role in HR-mediated repair of DSB [80,84,85,200]. In neurons produced from iPSCs that were reprogrammed from FAD patients, elevations of BRCA1 activity are associated with events of cell cycle progression (phosphorylation of CDC25C at serine 216) with concurrent increases in Aβ, suggesting an association of cell cycle re-entry with the utilization of HR in post-mitotic neurons that are regenerated [89]. Cell cycle re-entry induces apoptosis in neurons [201].

In NSCs, Aβ_42_ oligomers (AβO) induces ROS and DSBs with concomitant impairment of NHEJ-mediated DSB repair; enhancing DNA-PK function protects NSCs from AβO-induced toxicity [202]. Taken together, evidence supports a need to maintain genome integrity in neuron regeneration, which plays an anti-AD role.

### 5.4. Contributions of Chromosome Instability to AD

Chromosome instability (CIN) is a typical outcome of defects in DDR and activates DDR [203,204]. Accumulative evidence reveals increases in somatic aneuploidy as a feature of aging [205]. The rate of mosaic CIN increases from 0.23% for cancer-free individual under 50 years to 1.91% for those with ages of 75-79 years [205]. Brain has a high level of aneuploidy and the frequency increases with aging [206]. CIN is associated with neurodegeneration [207]. X chromosome aneuploidy is associated with AD [207,208]. Down syndrome is associated with chromosome 21 trisomy and all DS patients develop AD [145,146,147,148]. The existence of the A4 gene encoding APP in chromosome 21 is likely a contributing factor for the high rate of AD in DS patients [209]. However, this does not exclude a major role of CIN-associated genome instability in AD pathogenesis; in addition to chromosome 21, AD has a higher rate of aneuploidy in chromosomes 18 and X [208,210].

## 6. Systemic Alterations of DNA Repair Genes in AD Patients

In addition to changes in genes functioning HR and BER in AD brains, polymorphisms in these genes have been detected in blood and are associated with AD risk [211,212]. Using peripheral blood mononuclear cells (PBMCs) isolated from AD patients (*n* = 22) and healthy individuals (*n* = 13), profiling of mRNA expression identified 593 differentially expressed genes (DEGs) in AD subjects with 428 DEGs upregulated and 165 DEGs downregulated. These DEGs are enriched in pathways regulating inflammation, DDR, cell cycle, and neuronal processes [211]. Interestingly, DNA lesions are elevated in PBMCs of AD patients [213]. Compared to control subjects (*n* = 40), γH2AX, indicative of DSBs, is increased in lymphocytes of AD patients [214]. The increase can stratify AD patients from control individuals with an area under the curve (AUC) value of 0.91, sensitivity of 0.85 and specificity of 0.92 [214], suggesting a potential biomarker value of DSB in AD diagnosis. Clearly, the results need to be confirmed, as the cohort used in this study is quite small. In a similar study, increases in γH2AX were observed in lymphocytes of MCI subjects compared to controls [215].

Consistent with BER playing an essential role in repair oxidative DNA damage in neurons of CSN, there are numerous investigation of changes in BER genes in the circulation of AD patients [216]. In a study of PBMCs from 105 AD patients and 130 controls, the polymorphisms of c.580C>T and c.1196A>G of XRCC1 and c.977C>G of OGG1 are significantly associated with AD risk (Table 2) [216]; both XRCC1 and OGG1 function in BER (Figure 3). In comparison to 110 controls, significant increases in 8-oxoG DNA content with concomitant downregulations of 8-oxoG DNA glycosylase OGG1 and MUTYH [217], other DNA glycosylase NEIL1, APE1, and PARP1 were demonstrated in the PBMCs of AD patients (*n* = 100) (Table 2) [218,219]. Elevations in serum 8-OHdG was reported in AD patients (*n* = 30) compared to age-matched controls (*n* = 30) [220]. Polymorphisms of XRCC1 rs25487 (c.1196A > G; https://www.ncbi.nlm.nih.gov/snp/rs25487) (odds ratio/OR 3.72, 95% confidence interval/CI 1.739-7.891) and PARP1 rs1136410 (OR 4.159, 95% CI; 1.978−8.745) are significantly associated with AD risk [212]. The expression of the catalytic subunit POLD1 of DNA polymerase δ is significantly reduced in PBMCs of AD patients (*n* = 60) compared to controls (*n* = 40) (Table 2) [221]. Decreases in the expression of these BER genes in PBMCs of AD patients (Table 2) are not likely due to promoter methylation. In a study of LOAD patients (*n* = 56) and controls (*n* = 55) using PBMCs, no difference in promoter methylation was detected for OGG1, PARP1, BRCA1, MRELLA, MLH1, and MGMT [222]. Collectively, evidence supports biomarker values of alterations of BER genes in AD diagnosis (Table 2).

## 7. Conclusions

AD, particularly sporadic LOAD, is a multi-factorial disease, including metabolic dysfunction, insulin resistance, and others [223]. Among these factors, DNA damage is clearly an important one. In view of the typical AD pathology of Aβ-based senile plaques and hyperphosphorylated tau-formed neurofibrillary tangles, both Aβ peptides and tau affect genome instability. For example, nuclear tau plays a role in maintaining pericentromeric heterochromatin (PCH); loss of tau disrupts PCH, leading accumulation of DNA breaks [224]. Tau aggregation in the cytosol will deplete the nuclear pool of tau, abolishing tau-derived protection of genome integrity [225]. This concept is supported by the inability of hyperphosphorylated tau in the protection of DNA from thermal denaturation [226] and the loss of heterochromatin in tau transgenic Drosophila and mice, which contributes to neurodegeneration [227]. These associations of Aβ peptides and tau pathology with DNA damage clear strengthen the importance of DNA damage in AD pathogenesis. Accumulative evidence reviewed here collectively reveals the critical contributions of abnormalities in DNA lesion repair for both DSB and SSB in AD pathogenesis. In addition to neurons, DNA damage also occurs in oligodendrocytes, which has a major impact on AD [228,229]. Oligodendrocytes are exclusively responsible to produce myelin ensheathing axons of CNS [230,231]. Myelination is essential for the high speed transmission in the neural network; myelinated fibers have at minimum 10-fold faster conduct velocity than unmyelinated fibers with the same diameter [232]. Loss in myelination impairs performance of CNS, leads to neurodegeneration, and is among the earliest abnormalities during AD pathogenesis (see review [233]). Oligodendrocytes are vulnerable to oxidative DNA damage, which contributes to loss of neurons and onset of AD (see review [228]). This concept is in accordance with the observed declines of brain white matter, of which myelin constitutes 50%–60% [234], during aging [235,236].

## 8. Future Perspectives

For many years, AD research and drug development have been largely focused on the amyloid-cascade hypothesis which places Aβ at the apical position in AD pathogenesis. Because of fails in all phase 3 clinical trials on drug targeting the Aβ process, it is apparent that the hypothesis misses some major components particularly for LOAD cases. For instance, tau pathology seems to show higher match with AD development than with Aβ pathology [31]. Based on a study using human NSC line hNS1, Aβ42 at low concentration promotes hNSCs proliferation without compromising neuronal differentiation [237]. DNA damage clearly is a major contributing factor to AD. The pathogenicity of DNA damage is not only strengthened by its association with the typical AD pathological factors (Aβ peptide and tau pathology, see Section 7) but also impairment of DNA damage repair occurs prior to the onset of AD [55,78,79,238].

Considering aging being the strongest risk factor of AD and accumulative DNA damage as a well-established influence on aging, the concept of DNA damage abnormalities as the major contributor of AD is appealing. Although this concept is supported by a large amount of preclinical and clinical evidence as reviewed here, the pathological cause of DNA damage in AD remains to be demonstrated. This task is not only challenging but also should be cautiously considered. AD is a progressive neurodegenerative disease and likely also a systemic disease, in which abnormalities in DNA damage are an aspect. Nonetheless, evidence supports a major contribution of DSB accumulation in AD-associated loss of memory and neuron; for instance knockdown of BRCA1 in mice causes DSB, loss of neurons, as well as deficits in learning and memory [239]. Additionally, DSBs may occur prior to the onset of AD [78,79]. In view of AD being a progressive neurodegenerative disease, the current knowledge indicates a utility of early disease intervention through prevention or reduction of DSBs or other type of DNA damage. This strategy could delay the systemic alterations that progressively occur during AD development.

The common detection of alterations of the BER genes in circulation (Table 2) and their potential in stratification of AD risk support their diagnostic applications. Although more research is clearly needed for these applications, their potential should certainly be carefully investigated, particularly considering the lack of biochemistry-based means in current AD diagnosis (https://www.mayoclinic.org/diseases-conditions/alzheimers-disease/diagnosis-treatment/drc-20350453) and the non-invasive nature of obtaining PBMCs. The presence of alterations of the BER genes in circulation is in line with the hypothesis of AD being a systemic disease. The concept of AD, particularly LOAD cases (the vast majority of AD), as a systemic disease is consistent with DNA damage being a major factor of aging. In this regard, there is a need to develop a bona fide animal model for aging in which DNA damage accumulates in the brain during the aging process in order to systemically study AD. Owing to its unique anti-aging function, SIRT6-based animal models will be appealing. The mutation rate increases following aging in mouse liver but not in the animal brain [40], thus mouse models of AD likely have major limitations in studying aging process of LOAD.

## Figures and Tables

**Table 1:** Downregulation of BER in Alzheimer’s disease (AD).

Factors	Observation	Cohort (n)	Ref.
DNA glycosylase	Activity decrease in AD	20	[118,119]
OGG1	Deletion of codon C796; loss of 8-oxoG DG activity	2/14	[121]
OGG1	A53T and A288V; significant reduction in 8-oxoG DG activity	1/14 for each mutation	[121]
NEIL1	Decrease protein expression in AD	6	[120]
PARP1	Activity increase in AD	20	[129]
POLβ	Downregulation	10	[119]

OGG1: DNA glycosylase; NEIL1: Nei like DNA glycosylase 1; PARP1: poly(ADP) ribose polymerase 1; POLβ: DNA polymerase β.

**Table 2:** Alteration of BER genes in PBMCs of AD patients.

BER Genes	Changes^i^	AD Patients	Controls	Ref
OGG1	Reduction c.977C>G	*n* = 100 *n* = 105	*n* = 110 *n* = 130	[218,219] [216]
MUTYH	reduction	*n* = 100	*n* = 110	[219]
NEIL1	reduction	*n* = 100	*n* = 110	[219]
APE1	reduction	*n* = 100	*n* = 110	[219]
PARP1	Reduction rs1136410	*n* = 100 *n* = 120	*n* = 110 *n* = 110	[219] [212]
XCCR1	c.580C>T, c.1196A>G rs25487 (c.1196A>G)	*n* = 105 *n* = 120	*n* = 130 *n* = 110	[216] [212]
POLD1	reduction	*n* = 60	*n* = 40	[221]

i: AD vs control; AD: Alzheimer’s disease; APE-1: AP (apurine/apirimidine) endonuclease 1; OGG1: DNA glycosylase; MUTYH: MYH glycosylase; NEIL1: Nei like DNA glycosylase 1; PARP1: poly(ADP) ribose polymerase 1; POLD1: the catalytic subunit of DNA polymerase δ; XRCC1: X-ray repair cross-complementing protein 1.

## Abbreviations

8-OHdG	8-hydroxy-2’-deoxyguanosine
8-OHG	8-hydroxyguanine
AD	Alzheimer’s disease
Aβ	amyloid-β
AICD	APP intracellular domain
APE-1	AP (apurine/apirimidine) endonuclease 1
APP	amyloid precursor protein
APTX	ataxia with oculomotor apraxia
ATM	ataxia-telangiectasia mutated
AUC	area under the curve
BER	base excision repair
CDK	cyclin-dependent kinase
CIN	Chromosome instability
CNS	central nervous system
CSF	cerebrospinal fluid
DEGs	differentially expressed genes
DSBs	double strand breaks
FEN1	FLAP endonuclease 1
FXTAS	fragile-X-associated tremor/ataxia syndrome
HR	homologous recombination
HSV-1	herpes simplex virus-type 1
iPSC	induced pluripotent stem cells
LOAD	sporadic late onset AD
MCI	mild cognitive impairment
MUTYH	MYH glycosylase
NDP	adenine dinucleotide
NHEJ	non-homologous end joining
NEIL1	Nei like DNA glycosylase 1
NTH1	Nth like DNA glycosylase
OGG1	DNA glycosylase
PARP1	poly(ADP) ribose polymerase 1
PBMCs	peripheral blood mononuclear cells
PCAD	preclinical AD
PNKP	polynucleotide kinase phosphatase
POLβ	DNA polymerase β
POLD1	the catalytic subunit of DNA polymerase δ
PP4	Protein phosphatase 4
ROS	reactive oxygen species
SSBs	single strand breaks
TDP1	tyrosyl-DNA phosphodiesterase 1
TOP2β	topoisomerase Iiβ
WHO	World Health Organization
XRCC1	X-ray repair cross-complementing protein 1.
